# Anaerobic Biodegradation of Detergent Surfactants 

**DOI:** 10.3390/ma2010181

**Published:** 2009-03-16

**Authors:** Ute Merrettig-Bruns, Erich Jelen

**Affiliations:** Fraunhofer Institute for Environmental, Safety and Energy Technology UMSICHT, Osterfelder Str. 3, 46047 Oberhausen, Germany; E-Mail: erich.jelen@umsicht.fraunhofer.de

**Keywords:** Detergent surfactant, anaerobic biodegradation, test systems.

## Abstract

Detergent surfactants can be found in wastewater in relevant concentrations. Most of them are known as ready degradable under aerobic conditions, as required by European legislation. Far fewer surfactants have been tested so far for biodegradability under anaerobic conditions. The natural environment is predominantly aerobic, but there are some environmental compartments such as river sediments, sub-surface soil layer and anaerobic sludge digesters of wastewater treatment plants which have strictly anaerobic conditions. This review gives an overview on anaerobic biodegradation processes, the methods for testing anaerobic biodegradability, and the anaerobic biodegradability of different detergent surfactant types (anionic, nonionic, cationic, amphoteric surfactants).

## 1. Introduction 

Detergents are substances or preparations containing soaps or other surfactants intended for water-based laundry or dishwashing processes [1]. Detergents may be used in any form (liquid, powder, paste, bar, cake, molded piece, shape, etc.), widely for household laundry products, domestic and industrial cleaners, cosmetic products, and industrial purposes.

Surfactants are organic substances, used in detergents, intentionally added to achieve cleaning, rinsing and/or fabric softening due to its surface-active properties [2,3]. They consist of one or more hydrophilic and hydrophobic groups of such nature and size that they are capable of forming micelles [4]. Surfactants belong to a group of chemicals of high environmental relevance due to their large production volumes. They are mainly discharged into the environment by the wastewater pathway, either after treatment in a wastewater treatment plant or directly where no treatment system is available. Environmental compartments which may be influenced by surfactants are the freshwater environment (water body and sediment), the soil if surfactant-loaded sewage sludge is added, and the marine environment.

Biodegradation is an important factor for reduction and removal of organic contaminants from the environment. The evaluation of biodegradability of anthropogenic organic substances is an essential parameter for environmental risk assessment and required according to appropriate legislation [5]. The natural environment is predominantly aerobic, which for a long time has led to a focus on the biodegradation behavior of chemicals under aerobic conditions. Thus, a number of international recognized standard test methods regarding the aerobic biodegradability of substances have been developed. 

Nevertheless there are a few environmental compartments with absence of free oxygen and more or less anaerobic conditions such as river sediments and sub-surface soil layers, as well as anaerobic sludge digesters of wastewater treatment plants. In most cases a chemical’s environmental fate is accompanied by changing environmental conditions, e.g. transportation from aerobic to anaerobic zones. But it can not be excluded that chemicals such as surfactants which are released into the environment in relevant amounts may enter anaerobic compartments to a significant extent. 

The anaerobic biodegradability of chemicals so far has been considered to a minor extent, but its relevance is still in discussion [6]. In detergent regulation EC 907/2004 anaerobic biodegradability is not required for surfactants, but in the more stringent eco-labels this is a criterion [7]. Eco-labels are voluntary actions used in environmental policy with a precautionary approach which go further than existing legislation. The eco-labeling criteria usually cover limits for the toxicity of the ingredients and requirements for aerobic and anaerobic biodegradability.

Since 1988 standardized test methods for the determination of the ultimate anaerobic biodegradability of organic compounds are available [8,9]. But the data base for related tests with surfactants still is small compared to results from tests for aerobic biodegradability.

## 2. Characterization of surfactants 

**Figure 1:**
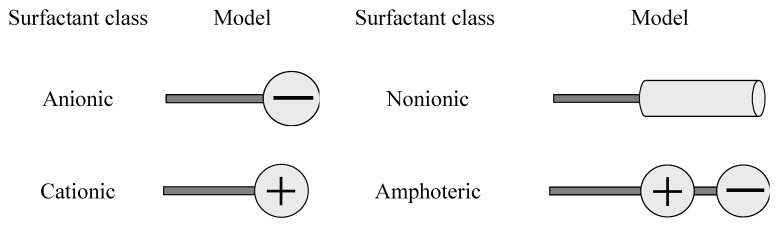
Surfactant classes [10].

Surfactants can be described with the tail-head model: the tail symbolizes the hydrophobic group, and the head the hydrophilic group. Because detergent surfactants are mainly used in aqueous systems surfactants are classified by the chemical structure of the hydrophilic group which is present after dissociation in aqueous solution (Figure 1).

### 2.1. Anionic surfactants

Most detergents contain a large amount of anionic surfactants. These surface-active compounds show anionic groups in their hydrophilic part and have a small counter ion, e.g. sodium or potassium, which has only minor influence on the properties of the substance [11]. The most important anionic surfactants used in laundry detergents are

- Soaps
- Alkylbenzene sulfonates (mainly LAS)
- Alkyl sulfates (AS)
- Alkyl ether sulfates (AES)
- Secondary alkane sulfonates (SAS)

Soap has remained the largest surfactant by volume world-wide. It is still the surfactant of choice in many countries. A decreased use of soap can be found in laundry detergents, due to its sensitivity to water hardness. The primary function remaining for soap in Europe is as a foam regulator in laundry detergents.

In Europe, USA, and Japan soap has been largely replaced by the synthetic anionic surfactants LAS [11]. Linear LAS show very good detergency performance and, as a result of their high solubility, LAS are also frequently used in formulations for liquid detergents. Like soap LAS are sensitive to water hardness [12]; the detergency performance of LAS is reduced with increasing water hardness.

AS are mainly alcohol sulfates and are produced either from natural fatty alcohols or from petrochemical substances. Their use has increased especially in concentrated products.

Alkyl ether sulfates are ethoxysulfates having a hydrophobic alkyl and a hydrophilic ethoxy chain. AES show a low sensitivity to water hardness in comparison to alkyl sulfates, as well as high solubility and good storage stability at low temperature in liquid formulations. Commercial AES consist of alkyl ether sulfates and alkyl sulfates as the main components. AES lead to intensive foaming and are thus well suited for the use in high-foam detergents for vertical-axis washing machines. Because of their specific properties AES are preferred constituents of detergents for delicate or wool washables, as well as foam baths, hair shampoos, and manual dishwashing agents. 

SAS are seen as special anionic surfactants for consumer products. SAS include high solubility, fast wetting properties, chemical stability and they are very similar to LAS in terms of detergency properties and water hardness sensitivity. They are completely insensitive to hydrolysis, even at extreme pH values, a result of the presence of the stable carbon–sulfur bond.

### 2.2. Nonionic surfactants

The share of nonionic surfactants in overall surfactant production and use has been increasing steadily since the 1970s [3]. The major contributors to this increase have been ethoxylates of fatty alcohols, oxo-alcohols, and secondary alcohols obtained by reaction of the corresponding alcohols with ethylene oxide. The higher use of nonionic surfactants in detergent formulations has partly been concomitant with the trend to wash at lower temperatures and with changes in the production shares of different fibers. The most important nonionic surfactants are:

- Alcohol ethoxylates (AE)
- Alkylphenol ethoxylates (APE)
- Fatty acid alkanolamides (FAA)
- Alkylamine oxides (AO)
- *N*-Methylglucamides (NMG)
- Alkylpolyglycosides (APG)

AE are the most important nonionics in detergent formulations. By varying the length of carbon chain and the degree of ethoxylation, these nonionic surfactants can be tailor-made with respect to the washing temperature.

APE are based on *p*-octyl-, nonyl-, and dodecylphenol poly(ethylene glycol) ethers. They achieved an early success due to their exceptional detergency properties, particularly their oil and fat removal characteristics. The usage of APE has largely declined, especially in Europe since 1986, due to a self-obligation of the industries to abandon their use. Their low biodegradability and the fish toxicity of certain metabolites resulting from partial biodegradation caused considerable environmental problems [13].

FAA alone have little application in laundry detergents. Their most important feature is foam boosting, i.e. adding desired stability to the foam produced by detergents prone to heavy foaming. This property is not desirable for horizontal-axis drum-type washing machines employed, e.g., in Europe. Nevertheless, small amounts of FAA as co-surfactants are capable of enhancing the soil removal properties of the classical detergent components at low washing temperatures. 

AO are produced by oxidation of tertiary amines with hydrogen peroxide. They show cationic behavior at acidic conditions and behave as nonionic surfactants at neutral or alkaline conditions. Despite good detergent properties, they are rarely included in laundry detergent formulations. The reasons for this are high costs and low thermal stability. 

NMG are a new type of nonionic surfactants that has been introduced in detergents in the 1990s. They are increasingly used as co-surfactants in powder and liquid detergent formulations.

APG consisting on an alkyl chain (hydrophobic) and sugar derivates (hydrophilic) have distinct lathering characteristics, especially in combination with anionic surfactants [14,15]. Due to their good foaming properties APG are predominantly used in dishwashing detergents, liquid detergents, and special detergents for fine fabrics. Since APG are completely based on natural resources, they ultimately biodegrade to carbon dioxide and water under aerobic conditions. 

### 2.3. Cationic surfactants

Cationic surfactants in detergent formulations are used as fabric softener in washing processes [3]. The most important ones are quarternary nitrogen compounds [16]:

- Mono- or di-alkyl quaternary compounds (DTDMAC)
- Esterified mono- or di-alkyl quaternary compounds (esterquats)
- Imidazoline derivatives

The first surfactant developed in this category was DTDMAC, introduced in 1949 as a fabric softener for cotton diapers and presented to the U.S. market as a laundry rinse-cycle fabric softener one year later. DTDMAC was the most important fabric softener for a long time. 

In the new generation of fabric softeners that came up in the 1980s/1990s, DTDMAC has been largely replaced by esterquats [17]. Due to their ester bonds which are potential breaking points, esterquats are readily biodegradable in contrast to DTDMAC. Esterquats possess favorable ecotoxicological and toxicological properties. 

Alkylated imidazoline derivatives are also used as fabric softeners and, due to dermatological compatability, as body care products.

### 2.4. Amphoteric surfactants

Amphoteric surfactants possess both anionic and cationic groups in the same molecule even in aqueous solution [3,18]. Despite excellent detergent properties, these surfactants are only rarely employed in laundry detergents, primarily for cost reasons. Among amphoterics, the betains are of economic importance. Betains are insensitive to water hardness, are only slightly toxic and compatible with the skin. They are mainly used in manual dishwashing and body care products. The most important types of amphoterics are:

- Alkyl betaine
- Alkylamidopropyl betaine
- Betaines derived from imidazolines
- Alkylamphoacetates

## 3. Surfactant production and use in Europe

**Figure 2:**
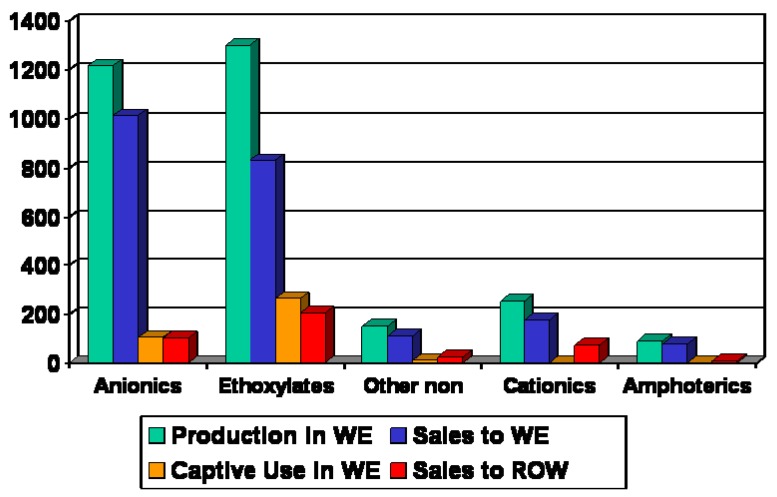
Surfactants 2007 in Western Europe (WE) and Rest of the World (ROW): 3 mio tons [19].

Annual statistics on the production and consumption of surfactants in Europe are provided by CESIO–Comité Européen des Agents de Surface et Leurs Intermédiares Organiques. The surfactants are not only used in the detergent industry but in other fields such as cosmetics, metal working, paper and leather industry. Half of the total surfactant consumption belongs to household application which is the largest market for surfactants. A brief summary of the surfactant statistics in Europe from 2007 is shown in Figure 2.

Most produced surfactants belong to the anionic and nonionic group, together they cover nearly half of the production volume. The other surfactant types are produced with much lower volumes. The nonionics, especially the ethoxylates, have passed the anionics in production volume and captive use.

## 4. Biodegradation

Biodegradation means the microbial degradation of organic substances. Depending on the degradation result, biodegradation with respect to surfactants is defined as follows [5]: 

*Primary biodegradation* means the structural change (transformation) of a surfactant by micro-organisms resulting in the loss of its surface-active properties due to the degradation of the parent substance and consequential loss of the surface-active property.

*Ultimate biodegradation* means the level of biodegradation achieved when the surfactant is totally used by micro-organisms resulting in its breakdown to inorganic end-products such as carbon dioxide, water and mineral salts of any other elements present (mineralization) and new microbial cellular constituents (biomass).

*Ready aerobic biodegradability* is an arbitrary classification of surfactants which have passed certain specified screening tests for ultimate biodegradability; these tests are so stringent that it is assumed that such compounds will rapidly and completely biodegrade in aquatic environment under aerobic conditions.

In opposite to former Detergent Guidelines which only required a primary biodegradability of anionic and nonionic surfactants the actual EU legislation prescribes the ultimate aerobic biodegradability of all surfactant types to be used in the detergent industry. The biodegradability under anaerobic conditions is not required in detergent regulation (648/2004) but it is a criterion in different European eco-labels [10]. Data on toxicity and biodegradability of surfactants have been collected in the Detergent Ingredient Database (DID-list) [20]. Anaerobically biodegradable surfactants are included on the DID-list. 

### 4.1. Anaerobic biodegradation pathway

**Figure 3:**
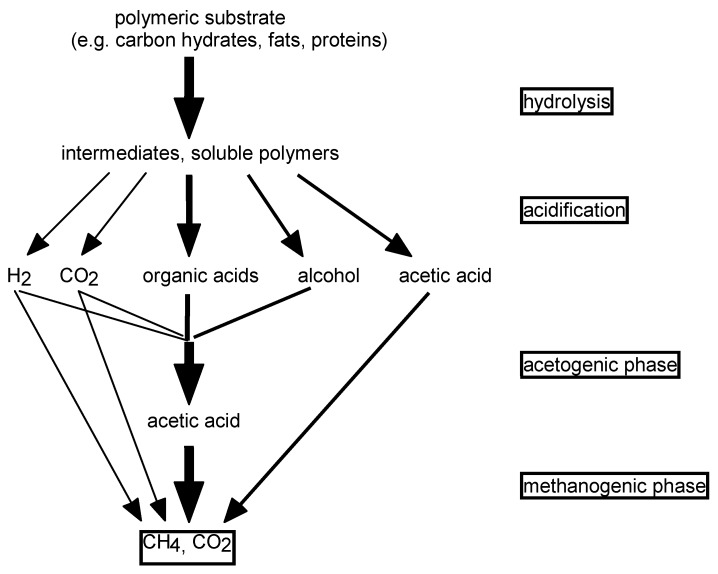
Anaerobic biodegradation pathway [21].

Anaerobic biodegradation means the microbial degradation of organic compounds under conditions free of molecular oxygen. In opposite to aerobic biodegradation pathways, where organic compounds often are mineralized by one type of microorganisms, the anaerobic biodegradation of a substance up to inorganic end-products always requires the co-operation of different types of microorganisms. The mixed culture works like a food chain, where the produced metabolites of one organism are the substrate for the next one (In the first step, complex or polymeric organic compounds are utilized by fermentative bacteria. Products of hydrolysis and acidification are metabolites of low molecular weight such as alcohols and short-chain fatty acids (C2–C4 organic acids). Acetogenic bacteria subsequently utilize these fermentation products as substrate and transform them to acetate, carbon dioxide and molecular hydrogen. At the end of the food chain, the methanogenic bacteria use acetic acid, carbon dioxide and hydrogen for the production of biogas–a mixture of methane and carbon dioxide. Carbonate can be used as hydrogen acceptor. Methanogenic bacteria are often the bottleneck of anaerobic biodegradation processes due to slow growth rates. These bacteria additionally are very sensitive against acidic condition; the optimal range of pH is 7–8. At pH lower than 6.5, biogas production is inhibited. Another bottle neck of anaerobic biodegradation may be the first reaction step (hydrolysis and acidification), especially if organic compounds of low bioavailability are used (Figure 3).

In the first step, complex or polymeric organic compounds are utilized by fermentative bacteria. Products of hydrolysis and acidification are metabolites of low molecular weight such as alcohols and short-chain fatty acids (C2–C4 organic acids). Acetogenic bacteria subsequently utilize these fermentation products as substrate and transform them to acetate, carbon dioxide and molecular hydrogen. At the end of the food chain, the methanogenic bacteria use acetic acid, carbon dioxide and hydrogen for the production of biogas–a mixture of methane and carbon dioxide. Carbonate can be used as hydrogen acceptor. Methanogenic bacteria are often the bottleneck of anaerobic biodegradation processes due to slow growth rates. These bacteria additionally are very sensitive against acidic condition; the optimal range of pH is 7–8. At pH lower than 6.5, biogas production is inhibited. Another bottle neck of anaerobic biodegradation may be the first reaction step (hydrolysis and acidification), especially if organic compounds of low bioavailability are used. 

In the presence of relevant amounts of nitrate and sulfate, alternative biodegradation pathways may occur like denitrification and sulfate-reducing processes, where nitrate and sulfate serve as hydrogen acceptors instead of carbonate (anoxic reactions). In marine sediments, the sulfate-reducing process is the predominant biodegradation pathway compared to methanogenesis. In contrast to the obligate anaerobic methanogenic and sulfate-reducing bacteria, the nitrate-reducing bacteria in general are facultative anaerobic, meaning that they are able to grow under aerobic and anaerobic conditions.

### 4.2. Factors influencing biodegradation

The biodegradation is influenced by several factors [21]:

- number of microorganisms capable of metabolizing the organic compound
- growth factors such as temperature, pH, nutrients, water content
- bioavailability of the organic substrate

For degradation of organic compounds at significant rates, an appropriate number of relevant microorganisms are needed. In biodegradation tests and technical biodegradation processes, the reaction can be started with an initial supply of microorganisms which are adapted to special conditions (e.g. aerobic or anaerobic) and/or to the special compound used as substrate source. During metabolization of the compound, the microorganisms proliferate and adapt to the special reaction conditions. Microbial biodegradation in continuously operated reactors can be considered as a self optimizing system. 

Convenient ambient conditions are a prerequisite for an optimal biodegradation process. Sufficient water content is the major factor for all biological processes. Temperature and pH are also important factors influencing microbial metabolism, and microorganisms differ greatly in their specific optimum in temperature and pH-value. Nutrition with macro and micro nutrients (e.g. trace elements and some vitamins) is needed to support optimal growth. If a complex organic substrate is used as reaction matrix, no further nutrition may be needed. Inhibition of microorganisms, which can be caused by the substrate itself or by degradation metabolites or products, may also occur. Most of the surfactants are known to cause microbial inhibition effects.

Reduced bioavailability of the organic compound is often a limiting factor in biodegradation processes. The bioavailability of an organic substrate mainly depends on its chemical fate, its dissolution rate and the mass transfer (e.g. from adsorbed to the aqueous phase). Surfactants tend to adsorb to solid particles, and some of the surfactants show rapid precipitation with water hardness ions such as calcium and magnesium [12]. Only water soluble molecules can be metabolized by microorganisms, so that biodegradation of adsorbed surfactants can be a function of the mass transfer rates rather than the degradation rates. The bioavailability of a compound, especially the adsorption behavior, has to be considered in a biodegradation test design.

## 5. Anaerobic environmental compartments

The biosphere is mainly aerobic. Anaerobic conditions in the environment occur where the oxygen consumption by biological oxidation processes exceeds the oxygen supply. This can either happen in small anaerobic sectors in an otherwise aerobic system, or in large and stable compartments such as marine or freshwater sediments, moorlands, and poorly drained soils. An overview and description on anaerobic compartments is given in [6].

### 5.1. Terrestrial compartments

Soils are typically aerobic systems, even though anaerobic micro sites may arise in poorly drained soils and may have a short depth of aerobic layer. As a general rule an anaerobic environment can be found in soils at a depth below 1 meter, the so called terrestrial subsurface. Moorlands are a typical anaerobic environment. High water content (> 80 %) leads to slow oxygen diffusion in soil pores. The oxygen is used by microorganisms which may cause oxygen deficiency. Flooding of soil may also lead to anaerobic conditions for a short time. Surfactants may reach the soil environment by application of surfactant loaded sewage sludge to agricultural land or landfill [22]. 

### 5.2. Aquatic compartments

Since nearly all the surfactants in household detergents go the (waste) water pathway, the aquatic environment is an important environmental compartment for potential surfactant pollution [6,23,24,25]. 

Freshwater environments include rivers with high exchange of water and lakes where water exchange may be limited by seasonal or permanent hydrographic conditions. In sediments with high oxygen consumption an anaerobic water body may arise. Freshwater lakes with depths greater than 10 m usually generate an anaerobic layer above the sediment. The largest anaerobic water bodies can be found in marine ecosystems such as the Black Sea being anaerobic from a depth of about 200 meters to 2,000 meters. Nevertheless the major decomposition of organic material in these environments (85–95%) is performed aerobically in the oxygenated zone. In marine sediments, sulfate-reducing bacteria dominate the anaerobic biodegradation processes.

Generally, lakes are much more sensitive to organic pollutant than rivers. Because of limited oxygen supply due to lower water exchange, discharge of waste water highly loaded with organic compounds into lakes results in fast oxygen consumption with subsequent anaerobic conditions. Sulfate-reducing processes result in production of hydrogen sulfide which affects higher organisms living in the lake.

In freshwater sediments the aerobic biodegradation is the predominant decomposition process. Influenced by season, organic load, water depth and flow, the sediment in rivers and lakes is usually anaerobic some mm below the surface. In rivers, the sediments are subject to dynamic processes involving sediment generation, transport, and erosion, influenced by several factors such as water flow rate, particle size of solid matter, and turbulent flow. Because of these processes, oxygen is brought to the sediments stimulating aerobic degradation processes. Lake sediments are less exposed to dynamic changes compared to river sediments which results in settlement of solid particles in much higher amount. If organic material is available in relevant concentration, microbial oxygen consumption effects broad anaerobic sediment zones. Compounds persistent under anaerobic conditions may be fixed in lake sediments and can be used as tracers to reconstruct the history of their release into the environment over some decades [26].

The aerobic zone of marine sediments can vary from a few mm in coastal areas to more than 1 m in deep sea sediments, depending on oxygen diffusion into sediment pore water, tidal flushing etc. In the anaerobic zones, the biological sulfate-reducing process is the predominant step in biodegradation of organic matter. Groundwaters may become anaerobic if they are contaminated with organic compounds which are biodegraded by aerobic bacteria. 

### 5.3. Wastewater treatment

In Europe about 80 % of waste water is treated in waste water treatment plants (WWTP) [27]. Therefore, the fate of surfactants in WWTP is of great importance. The biodegradation of organic waste water compounds in WWTP is usually performed under aerobic conditions but there are some special treatment steps working under anaerobic conditions such as:

- Mesophilic anaerobic digestion for sewage sludge stabilisation.
- Denitrifying process for elimination of nitrate (reduction to molecular nitrogen).
- Activated sludge systems with integrated oxygen limited zones for denitrifying processes with simultaneous aerobic/anaerobic conditions.
- Septic tanks which act as settling tanks for solids in domestic sewage that need to be periodically emptied periodically (e.g. once a year).

In the food processing industry, waste water with high organic pollution is often treated anaerobically. In most cases a post-aerobic treatment is used as a second step to reduce the anaerobic non-degraded residues including surfactants which may present.

## 6. Biodegradation Tests systems

### 6.1. Introduction

**Figure 4:**
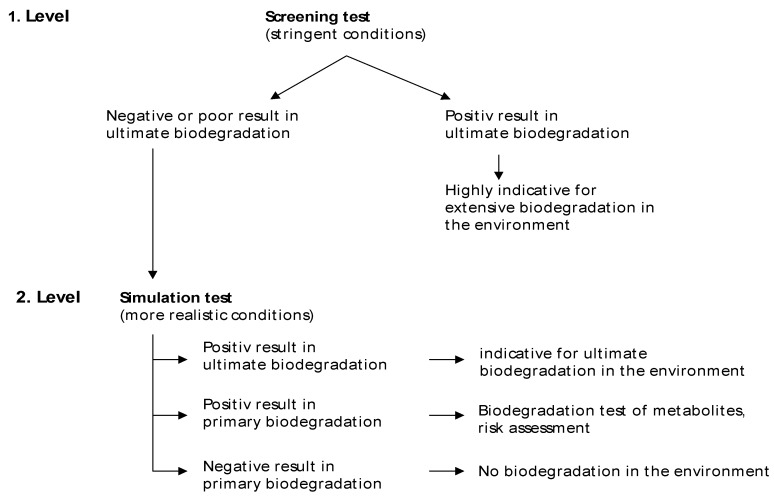
Feature of testing biodegradation (aerobic or anaerobic) [10].

Several systems for testing biodegradability are available [28,29]. Most of them have been developed for determination of aerobic biodegradability of substances and only a few for testing biodegradability under anaerobic conditions. In both cases it has to be distinguished between screening tests for determination of basic biodegradability under stringent conditions and test systems at simulation level for the assessment of biodegradability under more realistic conditions (Figure 4). On the first test level screening tests are performed. These are characterized by a simple test design (batch test) making them suitable for routine testing. The test conditions may differ considerably from realistic environment situations. It is a common feature of screening tests (aerobic and anaerobic) that they are more stringent (e.g. high test substance to biomass ratio) than the more realistic simulation tests. Positive degradation results of these tests are mainly independent on real environmental conditions and can be considered as highly predictive for good biodegradation of the tested compound in different environments. If positive results of ultimate biodegradation are achieved in screening tests, further testing is not necessary.

Otherwise, a poor degradation result in screening tests is not necessarily a proof of recalcitrance in real environment. In this case more representative tests under real-world conditions (simulation tests) should be performed on the second test level. Simulation tests are continuously working systems with more expenditure of test design, chemical analysis and test duration. Positive results of ultimate biodegradation in simulation test are indicators of ultimate biodegradation of the substance in the environment. If primary biodegradation is measured only, further assessment has to be made for study of possible metabolite behavior. If neither ultimate nor primary biodegradation is determined in the simulation test, this is highly indicative that the test substance is not degraded in the environment. A comparison of main characteristics of screening and simulation test is given in Table 1.

**Table 1:** Characteristics of screening and simulation test [10].

	Screening test	Simulation test
Test system	batch	continuous
Conditions	stringent	more realistic
Test design	simple	complex
Test substance	sole carbon source	together with (synthetic) sludge
concentration	high ratio concentration/biomass	low ratio concentration/biomass
supply	one-time supply	continuous supply
Expenditure	low	high
Test duration	30–60 days	min 70 days; residence time 20 days

Because of the complex test design, the high expenditure, and long test duration, simulation tests are not suitable for routine testing. Most of surfactants have been tested using screening tests.

### 6.2. Parameter of anaerobic biodegradation tests

Depending on analytical methods different biodegradation types can be evaluated. The different parameters describing anaerobic degradation are shown in Table 2. Specific analysis of the test substance is a parameter for determination of primary biodegradability. A decrease of test substance measured by specific analysis may be caused by several reasons and is not specific for biodegradation. Metabolites of the degradation process are usually not determined. The measurement of organic carbon content in liquid and solid phases covers degradation processes, adsorption, production of metabolites, and transformation of test substance in biomass. Additionally, carbon analysis in liquids and solids and calculation of carbon content in biogas allow a calculation of a carbon balance. Biogas production is a parameter of anaerobic mineralization, i.e. total degradation of test substance to inorganic end products. In such mineralization tests the ultimate biodegradability of an organic compound is determined. Therefore, testing mineralization is the most reliable method for the evaluation of biodegradation. 

**Table 2:** Parameters of anaerobic biodegradation [10].

Parameter	Kind of substance decrease
Specific analysis (decrease of test substance)	Primary degradation; mineralization, adsorption, non- determined metabolites, non-biotic removal, conversion to biomass
Dissolved organic carbon (DOC)	Mineralization, adsorption, conversion to biomass
biogas production	Mineralization

The required threshold limits of biodegradation depend on the kind of measured parameter. If primary biodegradation is tested, the threshold-pass level shall be 80 % or more. Measurement of organic carbon includes possible metabolites, the threshold limit is set at 70 %. Testing the mineralization by measuring the biogas production, the degradation rate is not referred to initial substance concentration but to calculated maximum theoretical biogas production. It has to be considered that these theoretical values are usually not reached since a relevant part of the test substance is transformed to biomass and not to biogas. The threshold limit in mineralization tests has been stated at 60 % [5].

### 6.3. Anaerobic screening tests

A lot of data are available about anaerobic biodegradability of surfactants [30]. Since 1988 standardized screening test methods for determination of the biodegradability of different organic compounds under anaerobic conditions have been available (Table 3).

All these tests build on the determination of biogas generation (manometric or volumetric measurement) as final product of anaerobic biodegradation process. The ultimate anaerobic biodegradability is determined. 

The first test method was the ECETOC test developed and published by the European Centre for Ecotoxicology and Toxicology of Chemicals in 1988 [33]. This method has been ring-tested and standardized as ISO 11734 [34] and OECD 311 [37]. The biogas production is measured manometrically. Pressure-resistant vessels are fitted with gastight septum. The increasing pressure caused by biogas generation is measured, e.g. by a pressure meter connected to a suitable syringe needle.

In ISO 14853 [36], a volumetric test system is described as an alternative method to the manometric one. The reaction vessel is connected to a graduated gas collecting tube, which is filled with acidified salt solution (barrier solution). The glass tube is connected via flexible gastight tubing to an expansion tank which is filled with barrier solution. Moving the tank up- and downwards, the solution surface of the tank can be adjusted to the one of the gas collecting tube and enable reading of gas volume at atmospheric pressure. 

In a round robin test, both ISO 11734 and ISO 14853 were verified using polymers as test substances [38]. Both methods were found to be suitable and practicable to perform anaerobic biodegradation screening tests.

**Table 3:** Standard screening tests for anaerobic biodegradation [31,32,33,34,35,36,37].

		Standards based on ECETOC		Test of Polymers		Polymers in high-solid matrix	
	ECETOC 1988	ISO 11734: 1995	OECD 311 2006	ISO 14853: 2005	ASTM D 5210-92 2007	ISO 15985: 2004	ASTM D 5511-02 2002
degradation parameter	Biogas, DIC in liquid phase	biogas in gas phase and soluble inorganic carbon (IC) in liquid phase	Biogas, DIC in liquid phase	biogas, CO_2_ and CH_4_ , DOC, TIC resp. DIC	biogas, CO_2_ and CH_4_ ,soluble organic carbon, residual polymer	biogas, disintegration of test substance, optional CO_2_ and CH_4_ in gas phase	biogas, CO_2_ and CH_4_ in gas phase
Test substance	div. material	soluble organic substance	div. material	non-soluble (polymeric) substance	polymer	non-soluble (polymeric) substance	polymer
medium	definite mineral salt medium	definite mineral salt medium	definite mineral salt medium	definite mineral salt medium	definite mineral salt medium	digested substance	digested substance
Test volume	100-1000 mL	100-1000 mL	100-1000 mL	250 mL	100 ml	ca. 1000 mL	ca. 1L
Test duration	8 weeks	60 d	60 d	30-60 d		15 d (or longer)	up to 70% degradation rate in reference substance
temperature	35 ° 2 °C	35 ° 2 °C	35°2 °C	35 ° 2 °C	35 ° 2 °C	52 ° 2 °C	52 ° 2 °C
method	manometric	manometric	manometric	volumetric or manometric	volumetric or manometric	volumetric (as example)	volumetric
concentration test substance	20-50 mg/L organic carbon	100 mg/L organic carbon	20–100 mg/L organic carbon	100 mg/L organic carbon		20 g DS with 8 g TOC /L	15-100 g dry substance /L
dry matter	1-5 g/L	1-3 g/L	1-3 g/L	1-3 g/L	1-2 g/L	> 200 g/L	>300 g/L

Most of the tests are screening methods for the evaluation of basic biodegradability in an aqueous medium. A definite mineral salt medium with volumes between 100–1,000 mL is used. Typical concentrations of test substance vary from 20–100 mg of organic carbon per liter. The amount of solids (biomass inoculum) is adjusted to 1-3 g of dry matter per liter, corresponding to an organic carbon to solids ratio ranged from 7 to 100 mg per g of dry matter. Blank controls without test substance are necessary to record the endogenous biogas production of the inoculum. To achieve a sufficient biogas production, which differs significantly from the blank control, a minimum test substance concentration of about 20 mg of organic carbon per g of solids is needed. A low test substance concentration is chosen when inhibition caused by the test substance is expected. The tests are incubated at mesophilic temperature (35 °C) and with test duration of 20 days up to 8 weeks. The tests are usually continued until a plateau phase is reached with no further gas generation. At the end of the tests the dissolved inorganic carbon (DIC) of the digestion liquid is determined and will be taken into account in calculation of the ultimate biodegradation rate of the test substance. 

The test methods working with high-solid systems [31,35] correspond to conditions in anaerobic dry digestion processes. Some of these screening tests have been developed for testing of packing and plastic material [31,32,35,36]. There are no special tests for determination of anaerobic biodegradability of surfactants so far. In the annex of detergent regulation 684/2004 and the eco-label systems, the ECETOC test method is proposed for determination of biodegradability under anaerobic conditions.

### 6.4. Anaerobic simulation tests

Simulation test systems are continuously running test systems which represent more realistic environmental conditions. They are specifically designed for different anaerobic environments and have a number of advantages compared to screening tests:

- realistic kinetic information of test substance biodegradation for different anaerobic environments using related inoculums,
- application of lower and more realistic test substance to biomass ratio which avoids inhibition caused by test substance,
- Acclimation of the microbial culture to the test substance during the continuous process.

Due to their complex test design, long test duration and expensive costs, simulation tests are not suitable as routine tests. These tests are more applicable for using as higher tier biodegradation tests, if the screening tests show poor degradation results making further testing necessary. Several simulation test systems have been described in publications [6,39]. Typical anaerobic bioreactors used for continuous operation are stirred tank systems [40], fixed bed reactors, and upflow sludge blanket (UASB) systems [13,41,42,43]. The latter two work with retention of the microbial biomass. Another system is a horizontal-flow immobilized biomass reactor (HAIB) which has been used to study the anaerobic degradation of anionic sulfonates [44]. 

Transformation tests of organic chemicals under aerobic-anaerobic conditions have been standardized by the Organisation of Economic Co-operation and Development-OECD (Table 4). In OECD 307 [45] the transformation is tested in soil, in OECD 308 [46] in aquatic sediment, respectively. The objective of these tests is to determine the rate of biodegradation of substances, to measure the rate and route of non-biotic and biotic transformation, and the distribution of transformation products between two phases (e.g. water and sediment). The application of ^14^C-labelled test material is usually required in these tests. Results from tests with surfactants have not been found.

**Table 4:** Standard simulation tests for anaerobic biodegradation [45,46].

	Aerobic and anaerobic transformation	
	OECD 307 2002	OECD 308 2002
degradation parameter	concentration of test substance and transformation product	concentration of test substance and transformation product
test substance	non-labelled or radiolabelled substance	non-labelled or radiolabelled substance
medium	soil	aquatic sediment
test volume	50–200 g soil (ds)	water/sediment-ratio: 3-4:1
test duration	max. 120 days	max. 100 days
temperature	20 ± 2 °C	10-30 °C
method	chemical analysis	chemical analysis
dry substance	40–60 % water hold capacity	min. 50 g

## 7. Anaerobic biodegradability of different types of detergent surfactants

A compilation of literature data on anaerobic biodegradation of surfactants of the different classes is given. Only results from the stringent screening tests are presented in the following tables. All data refer to ultimate anaerobic biodegradation based on measured biogas production. Results from simulation tests are discussed for surfactants proved to be poorly biodegradable in screening tests. Additionally, data of the Detergents Ingredients Database (DID list) are given as far as available.

### 7.1. Anionics

Most of data on anaerobic biodegradability have been found for anionic surfactants. Results of screening tests are shown in Table 5.

**Table 5:** Anaerobic biodegradability of anionics in screening tests (according ECETOC).

Surfactant type	Characterization	Test subst. in mg/L active matter	Test subst. in mg/L carbon	Inoculum in (dm) g/L	Test Duration in days	Result in %	References
**Soap**	Na-palmitate, Na-laurate, Na-stereate	70–1000		1–5	28-54	> 90	[47,48,49]
**LAS**	C_10_-C_13_	50		1-5	49	0	[50]
	C_8-12_		50		60	0	[51]
	C_10_-C_13_	151 75	100 50	1.5	84 119	0 0	[10]
	C_10_–C_14_		10-200	3–4.5	78	0	[52]
**SAS**	C_14_ +C_17_				17	0	[53]
	C_14_–C_17_		20-100	3	70	0	[54]
**Alpha-olefin sulfonates (AOS)**	C_14_–C_16_		20–100	3	70	0	[54]
**Methyl ester sulfonates (MES)**	C_10_–C_16_		20–100	3	70	0	[54]
**Dialkyl sulfo-succinates**	di-C_8_-SS		20-100	3	70	35–50	[54]
**Monoalkyl ethoxy sulfosuccinates**	C_12_-(EO)_3_-SS		20-100	3	70	23-> 80	[54]
**Alcohol Sulfates**	C_18_	50	29	3	56	88	[33]
	C_12_–C_18_	239	100	1.5	84	59	[10]
	C_12_–C_13_ linear	30		1-5	42	70	[55]
	C_14_–C_15_ 80 % linear	30		1–5	42	60	[55]
	C_12_–C_13_ mid-chain branched	30		1-5	42	40	[55]
	C_12_–C_13_ mainly branched	30		1–5	42	25	[55]
**Alkylether Sulfates Na salt**	C_12_		20	0.15	56	0-30	[56]
**Alcohol Ether Sulfate**	C_12-14_, 2 EO		50	1-5	41	75	[50]
	C_12_, xEO	40-100	20-50	0.06–0.12	55-56	14-41	[57]
	C_12-14_, 2 EO	191 95	100 50	1.5	84 119	0 60	[10]

Soaps are sodium/calcium/magnesium salts of natural fatty acids and have been found to be readily biodegradable under anaerobic conditions even in high concentrations up to 1000 mg/L covering different alkyl chain length (C_12_–C_18_). The biodegradability of soaps may be negatively influenced by poor bioavailability of Ca-, Mg-soaps. Soaps with C_12_–C_22_ alkyl chain are classified as anaerobic biodegradable on the DID list (no. 15).

Several screening and simulation tests have been performed with sulfonates, especially LAS. In contrast to the positive biodegradation results achieved under aerobic conditions, all published ECETOC test results show that none of the sulfonates are ultimately biodegradable under anaerobic conditions. In some simulation test systems high degradation rates of sulfonates were determined, even if these systems are not rigorously anaerobic but have oxygen-limited conditions [6,41,42,58]. Sulfonate biodegradation is initiated by aerobic or microaerophilic organisms and the metabolites may be degraded anaerobically. It seems that desulfonation of LAS at significant rates is only performed if oxygen is available. Monitoring studies performed in anoxic marine sediments indicate an anaerobic biodegradation of LAS in sulfate reducing environment [59]. The degradation intermediates sulpho phenyl carboxylic acids (SPC) have been detected in strictly anoxic zones [59,60]. The anaerobic biodegradation of LAS to SPC was confirmed in laboratory studies with anoxic marine sediments spiked with 10-50 ppm of LAS [59]. After 165 days, up to 79 % of LAS was degraded via the generation of SPC. The generation of mineralization products was not determined. Since the degradation rate was rather slow, its impact on anaerobic environmental fate of LAS is still unknown. Na-LAS salt has been found to inhibit biogas generation at concentrations of 5-10 g/kg dry sludge [52]. In real anaerobic environments such as sludge digesters sulfonates are not degraded significantly. Relative high amounts of sulfonates have been found in digested sludge with concentrations about 5,000 mg/kg dry sludge as a mean value [10]. Inhibition effects on biogas generation were not detected. Sulfonates may be present as poorly soluble Ca and Mg salts in sewage sludge resulting in less bioavailability and toxicity. Relevant LAS concentrations can be found in sludge amended soil immediately after sludge application. Several studies from different European countries show that LAS rapidly degrades in the predominant aerobic soil environment [10,61]. Sulfonates are classified as not anaerobic biodegradable on the DID list (no. 1–3, 13-14).

Linear alkyl sulfosuccinates are proven to be ultimately biodegradable under anaerobic conditions in concentrations up to 85 g/kg dry sludge [54]. The anaerobic biodegradation may be attributed to the presence of ester bonds whose cleavage does not need molecular oxygen. The anaerobic biodegradability of a branched alkyl sulfosuccinate was much lower with a maximum degradation rate of 50 %. Dialkyl sulfosuccinate was classified as not anaerobic biodegradable on the DID list (no. 10).

Linear alkyl sulfates proved to be ultimately biodegradable under anaerobic conditions in screening tests [10,62]. Alkyl sulfates with C_8_–C_18_ alkyl chain are classified as anaerobic biodegradable on the DID list (no. 4–7). Increased branching of the alkyl chain results in reduction of biodegradability [55]. 

The anaerobic biodegradability of alkyl ether sulfates seems to depend on test concentration. At low test concentrations of about 20–50 mg/L carbon alkyl ether sulfates are ultimately biodegraded under anaerobic conditions. At higher test concentrations or high test substance to biomass ratio the anaerobic biodegradability is inhibited. Alkyl ether sulfates with C_12_–C_18_ alkyl chains and 1–4 ethoxy units are classified as anaerobic biodegradable on the DID list (no. 8–9).

### 7.2. Nonionics

Most of the data on anaerobic biodegradability of nonionic surfactants have been found for alcohol ethoxylates and glucosides. Results of screening tests are presented in Linear alkyl ethoxylates are ultimately biodegradable under anaerobic conditions as demonstrated in several screening tests. The degradation has been reported in tests using different inoculums such as digested sewage sludge, freshwater sediment, and marine sediment [56]. The anaerobic biodegradability decreased with increasing branching degree of the alkyl chain and was improved by increased ethoxylation rate. Alkyl ethoxylates with linear C8–C18 alkyl chain and up to 30 ethoxy units are classified as anaerobic biodegradable on the DID list (no. 20–22, 24–25, 28–30, 34, 37–40) (Table 6). 

Linear alkyl ethoxylates are ultimately biodegradable under anaerobic conditions as demonstrated in several screening tests. The degradation has been reported in tests using different inoculums such as digested sewage sludge, freshwater sediment, and marine sediment [56]. The anaerobic biodegradability decreased with increasing branching degree of the alkyl chain and was improved by increased ethoxylation rate. Alkyl ethoxylates with linear C8–C18 alkyl chain and up to 30 ethoxy units are classified as anaerobic biodegradable on the DID list (no. 20–22, 24–25, 28–30, 34, 37–40). 

**Table 6:** Anaerobic biodegradability of nonionics in screening tests (according ECETOC).

Surfactant Type	Characterization	Test conc. In mg/L active matter	Test conc. Carbon in mg/L	Inoculum conc. Dm in g/L	Test duration in Days	Results in %	References
**Alcohol Ethoxylates**	C_9-11_, 8EO		20-50	0.15–1.5	56-96	>75	[56]
	Isotridecanol, (5,10,20) EO		20	2-3	110	0-30	[63]
	C_12_-C_15_, 7EO		20	0.15		35	[64]
	C_12-14_, (5,10,20) EO		20	2-3	110	29-94	[63]
	mono br C_14-15_, (10,20) EO		20	2-3	89	0-23	[63]
	Dehydol LT7	176	100	1.5	84	64	[10]
**Alkylphenol Ethoxylates**	Nonylphenol, 10 EO	50		1- 5	84	20,5 ± 12,6	[50]
	Nonylphenol, 9 EO	50		1	40-50	32-43 CH4	[65]
**Glucosides**	Ethyl 6-O-decanoyl glucoside		20		56	59-65	[57]
	APG (branched) C_8_, DP = 1,6	30-40	20	0.15		22	[64]
	APG (linear) C_12-14_, DP = 1,4	30-40	20	0.15		72	[64]
	C_12_-C_14_ APG		20	0.15	56-96	>75	[56]
	C_8_-C_14_ APG	100		3	56	>80	[66]
	Glucopon 215 CSUP	203	100	1.5	84	61	[10]
	C_12_ Ethylglucoside monoester	30-40	20	0.15	56-96	>75	[56]
	C_10_-C_12_ 6-O-Ethyl-glucoside monoester		20–50	0.15–1.5	56–96	>75	[56]
**Amine Oxides**	C_12_–C_14_		15–150	4.2–4.8	90	0	[67]
	Cocoamido-AO		35–275	4.2–4.8	90	> 70	[67]
**Alkylethanol-amides**	Cocomonoethanol-amide		20	1–3	56	81	[23]

DP = degree of polymerization.

Alkylphenol ethoxylates are proved to be poorly to moderately biodegradable under anaerobic conditions. In screening and simulation tests the metabolite nonylphenol accumulated [13,68]. This indicates that alkyl phenol ethoxylates are degraded via sequential removal of ethoxy units to the hydrophobic alkyl phenol. The same results were described for anaerobic biodegradation under denitrifying, sulfate-reducing, and iron(III)-reducing conditions [69,70,71]. The metabolite alkyl phenol is poorly degradable both under aerobic and anaerobic conditions and affects the aquatic environment due to its proven estrogenic activity [72].

Among the sugar derivatives, the alkylpolyglucosides (APG) and glucoside esters proved to be ultimately biodegradable under anaerobic conditions. The anaerobic biodegradability of APG decreased with increasing branching degree of the alkyl chain. The sugar derivative surfactants with linear alkyl chain are classified as anaerobic biodegradable on the DID list (glucose amides: no 45–46; APG: no 47, 49).

Only a few data are available for the biodegradability of amine oxides. Some results are presented in a recent publication [67]. C_12_-C_14_ alkyl amine oxides were not biodegraded under anaerobic conditions in a screening test. In contrast, former results from a simulation test using radiolabelled dimethyl dodecyl amino oxide indicate a high removal rate [73]. Nevertheless, C_12_-C_18_ alkyl amine oxide is classified as anaerobic biodegradable on the DID list (no. 62). In contrast to the alkyl amine oxides the cocoamido propyl amine oxide was ready and ultimately biodegraded under anaerobic conditions in the screening test. This amine oxide is not listed in the DID list so far. The difference in biodegradability may be attributed to different toxic effects of the compounds.

Cocomonoethanolamide was shown to be ultimately biodegradable under anaerobic conditions in a screening test. Coconut fatty acid monoethanolamide and its ethoxylated derivative are classified as anaerobically biodegradable on the DID list (no. 50 -51). 

### 7.3. Cationics

As mentioned above, cationic surfactants are used in much lower amounts in detergent formulations compared to anionic and nonionic surfactants. According to the detergent regulation 684/2004 cationic surfactants should be ultimately biodegradable under aerobic conditions. Only a few data are available on the anaerobic biodegradability. One problem in biodegradation tests is the inhibitory of cationics even at low concentrations [74,75,76]. Results of screening tests are shown in Table 7.

**Table 7:** Anaerobic biodegradability of cationics in screening tests (according ECETOC).

Surfactant Type	Characterization	Test conc. In mg/L active matter	Test conc. Carbon in mg/L	Inoculum conc. Dm in g/L	Test duration in Days	Results in %	References
**Alkyl quarternary ammonium compounds**	Cetyltrimethyl-ammonium bromide CTMAB (C_16_)	50	50	2-3	60	0 (inhibition)	[51]
	DHTDMAC	26–129	20–100	4.0	100	0	[77]
	Bis(acyloxyethyl)-hydroxyethyl-methyl ammonium-methosulfate BAHMA	77	50	1.5	120	24	[10]
**Esterquats**	MTEA	50		1–5 as dry matter	42	101.1 ± 12,8	[17]
	DEEDMAC (C_16-18_)	~ 38		1 to 5	60	90	[78]
	*N,N*-di-(β-acyloxy-ethyl),*N*-β-hydroxy-ethyl,*N*-methyl ammonium-methylsulfate	30–152	20–100	4.0	100	70–100	[77]
	quaternized fatty acid imidazoline methosulfate	73	50	1.5	120	64	[10]

Alkyl quarternary ammonium compounds such as DHTDMAC and CTMAB are shown as not ultimately biodegradable under anaerobic conditions in screening and simulation tests [74,75]. 

Esterified quarternary ammonium compounds such as esterquats have been proved to be ultimately biodegradable in anaerobic screening tests. Alkyl ester ammonium salts are classified as anaerobic biodegradable on the DID list (no. 71).

### 7.4. Amphoterics

Similar to cationics, the amphoteric surfactants are used in low amounts. Only a few data are available on anaerobic biodegradability. Results of screening tests are shown in Table 8.

**Table 8:** Anaerobic biodegradability of amphoterics in screening tests (according ECETOC).

Surfactant Type	Characterization	Test conc. In mg/L active matter	Test conc. Carbon in mg/L	Inoculum conc. dm in g/L	Test duration in Days	Results in %	References
**Alkyl betaines**	C_10_ C_12_ C_14_		50–100 20–200 50–100	2.8–3.4	60	0	[79]
	Coco betaine	239 120	100 50	1.5 1.5	84 119	0 0	[10]
**Alkylamido betaines**	Cocoamido propyl dimethyl betaine		30–300	2.8–3.4	60	>60	[79]
	Cocoamido propyl betaine	214 107	100 50	1.5 1.5	84 119	67 72	[10]
	Cocoamido propyl betaine		50		56	75	[23]
**Alkyl imidazoline derivatives**	C_10_ C_12_ C_14_		50–100 20–200 50–100	2.8–3.4	60	>60	[79]
**Alkyl amphoacetates**	Cocoampho-diacetate		9.5	1.1	56	79.8	[24]

Alkyl betaines with different alkyl chain length are proved to be not ultimately biodegradable under anaerobic conditions. This is a result of several screening tests covering test substance concentrations of 20–200mg/L carbon. Some primary biodegradation may occur under anaerobic conditions but no mineralization [10]. Results from simulation tests have not been found.

Alkylamido betaines are ultimately biodegradable under anaerobic conditions even at high concentrations up to 300mg/L carbon. At the highest test substance concentration an initial inhibition of the biogas production was observed for about four weeks [79]. After this adaption phase the biodegradation increased. Alkyl C_12_/C_18_ amidopropyl betaine is classified as anaerobic biodegradable on the DID list (no. 61).

Alkyl imidazoline derivatives are also shown as ultimately anaerobic biodegradable in screening tests covering test substance concentrations of 20–200 mg/L carbon. 

Cocoamphodiacetate was ultimately anaerobic biodegradable in a screening test at a rather low test concentration of <10 mg/L carbon. 

## 8. Conclusions

Surfactants used in detergent formulations are released in relevant amounts into the environment by the waste water pathway. According to the high environmental relevance surfactants have to meet certain requirements issued in the European detergent regulation 684/2004. All types of surfactants should be ultimately biodegradable under aerobic conditions, one of the most important mechanisms for removal of chemicals released into the environment. 

Anaerobic biodegradability is not required so far with except of eco-labelled products. Although the natural environment is predominately aerobic, there are some more or less strict anaerobic compartments such as river sediments, sub-surface soil layer and anaerobic sludge digesters of wastewater treatment plants. In these compartments anaerobic biodegradation may contribute to avoid accumulation of anthropogenic chemicals. 

For both testing biodegradability under aerobic and anaerobic conditions standardized test methods are available. Stringent screening tests to determine the anaerobic mineralization are usually chosen as a first step in the biodegradability test scheme. Surfactants poorly degradable in screening tests are often studied in more complex simulation tests.

Due to its higher environmental relevance most data are available for aerobic biodegradability of surfactants. In the last years the anaerobic biodegradability has been determined for an increasing number of surfactants covering all classes. Surfactant types with straight (C-C) alkyl chains seem to be less susceptible to be anaerobically biodegraded than other surfactants, unless in sulfate reducing environments. An integration of heterogeneous atoms or groups such as ester bonds improves the anaerobic biodegradability of such compounds significantly. 

Many surfactants of large production volumes used in detergent formulations belong to the anionic and nonionic group. With the exception of the anionic sulfonates most of these surfactants have been proved to be biodegradable under anaerobic conditions. Sulfonates are known to be aerobically mineralized only. 

In the last few years also cationic and amphoteric surfactants have been tested. Cationic surfactants are widely used as fabric softener in household laundry products and conditioner in cosmetic applications. The poorly aerobically biodegradable alkyl quaternary ammonium compounds have been largely replaced by the ready degradable esterquats. Esterquats are also ultimately biodegradable under anaerobic conditions. 

Amphoteric surfactants mainly consist of betaine derivatives. They are predominately used in personal care products and manual dishwashing products due to its high skin compatibility. In contrast to the not anaerobic biodegradable alkyl betaine the alkylamido betains were mineralized under anaerobic conditions.

Numerous data are available on aerobic biodegradability of surfactants which have been studied for several decades. A systematic study of the biodegradability under anaerobic conditions started about twenty years ago, but the data base covering all surfactant classes has increased especially over the last few years.

## Abbreviations

AE: Alcohol ethoxylates
AES: Alcohol ether sulfates
AO: Amine oxides
AOS: Alpha olefin sulfonates
APE: Alkyl phenol ethoxylates
APG: Alkyl polyglucosides
AS: Alcohol/alkyl sulfates
ASTM: American Society for Testing and Materials
BAHMA: Bis(acyloxyethyl) hydroxyethyl methyl ammonium methosulfate
CESIO: Comité Européen des Agents de Surface et leurs Intermédiares Organiques
CTEA: Cetyltriethylammonium bromide
CTMAB: Cetyltrimethylammonium bromide
DEEDMAC: Diethylester diethylammonium chloride
DHTDMAC: Di(hydrogenated tallow) dimethyl ammonium chloride
DID: Detergent Ingredient Database
dm: Dry matter
DTDMAC: Ditallowdimethylammonium chloride
ECETOC: European Chemical Industry Ecology and Toxicology Center
FAA: Fatty acid alkanolamides
ISO: International Organization for Standardization
LAS: Linear alkylbenzene sulfonate
MES: Methyl ester sulfonate
MTEA: Methyltrihydroxyethylammonium chloride
NMG: *N*-Methylglucamides
OECD: Organisation of Economic Co-operation and Development
SAS: Secondary alkane sulfonate
SPC: Sulphophenyl carboxylates
SS: Sulphosuccinates
UASB: Upflow anaerobic sludge blanket reactor
